# Charting the NF-κB Pathway Interactome Map

**DOI:** 10.1371/journal.pone.0032678

**Published:** 2012-03-05

**Authors:** Paolo Tieri, Alberto Termanini, Elena Bellavista, Stefano Salvioli, Miriam Capri, Claudio Franceschi

**Affiliations:** 1 CIG ‘Luigi Galvani’ Interdept Center, University of Bologna, Bologna, Italy; 2 Department of Experimental Pathology, University of Bologna, Bologna, Italy; 3 IAC-CNR Istituto per le Applicazioni del Calcolo, Consiglio Nazionale delle Ricerche, Rome, Italy; McMaster University, Canada

## Abstract

Inflammation is part of a complex physiological response to harmful stimuli and pathogenic stress. The five components of the Nuclear Factor κB (NF-κB) family are prominent mediators of inflammation, acting as key transcriptional regulators of hundreds of genes. Several signaling pathways activated by diverse stimuli converge on NF-κB activation, resulting in a regulatory system characterized by high complexity. It is increasingly recognized that the number of components that impinges upon phenotypic outcomes of signal transduction pathways may be higher than those taken into consideration from canonical pathway representations. Scope of the present analysis is to provide a wider, systemic picture of the NF-κB signaling system. Data from different sources such as literature, functional enrichment web resources, protein-protein interaction and pathway databases have been gathered, curated, integrated and analyzed in order to reconstruct a single, comprehensive picture of the proteins that interact with, and participate to the NF-κB activation system. Such a reconstruction shows that the NF-κB interactome is substantially different in quantity and quality of components with respect to canonical representations. The analysis highlights that several neglected but topologically central proteins may play a role in the activation of NF-κB mediated responses. Moreover the interactome structure fits with the characteristics of a bow tie architecture. This interactome is intended as an open network resource available for further development, refinement and analysis.

## Introduction

### Inflammation, a complex, pervasive process

Inflammation is a complex, systemic, multi-scale physiological process necessary to cope with damaging agents and fundamental for survival, involving a variety of cells, organs and organ systems. The complexity of the inflammatory process has escaped reductionist and linear approaches, since it is characterized, among other features, by non-proportional kinetics as well as numerous and nested feedback loops [1]. Apart from its protective, physiological role, inflammation is an important concomitant cause of many major age-associated pathologies, such as cancer, neurodegeneration, Type II Diabetes and metabolic syndromes [2]. To this regard, it has been observed that a systemic, chronic, low-grade inflammatory status characterizes the aging process, and that markers of inflammation increase with age [3]–[7]. We proposed to indicate this phenomenon as “inflammaging” [8]–[10]. In this perspective, we hypothesised that the phenomenon of inflammaging, likely resulting from the chronic exposure to environmental stressors such as chronic viral infections [6], [11], can be an important cause of these age-associated diseases, and therefore the genetic control of inflammation appears to be a crucial determinant of longevity, today a pressing topic given the aging of population all over the world.

A pivotal player of the inflammatory response is the NF-κB (nuclear factor kappa-light-chain-enhancer of activated B cells) transcription factor. Able to be activated by a great number of stimuli and to participate in the regulation of hundreds of genes [12], [13], NF-κB has a wide spectrum of actions, such as to induce survival and proliferation, and in particular it is considered *the* master regulator of inflammation [14]. Thus, it is of great interest to deepen the basic knowledge regarding the topology and the dynamics of the signaling pathways and regulatory network underpinning the activation of such a pleiotropic transcription factor. While the literature dedicated to specific aspects of NF-κB functions or regulation is huge, a wider representation of its complex regulating system is still missing.

### The NF-κB system

NF-κB is a protein complex that both induces and represses gene expression by binding to discrete DNA sequences, known as κB elements, in gene promoters and enhancers [15], [16]. In mammalian cells, there are five NF-κB family members, RelA (p65), RelB, c-Rel, p50/p105 (NF-κB1) and p52/p100 (NF-κB2), and different NF-κB complexes are formed from their homo- and hetero-dimers. All proteins of the NF-κB family share a Rel homology domain. Moreover, RelA, RelB, and c-Rel have a transactivation domain in their C-terminus required for gene activation. NF-κB 1 and NF-κB 2 proteins are synthesized as large precursors, p105 and p100, which undergo processing to generate the mature NF-κB subunits, p50 and p52, respectively. The processing of p105 and p100 is mediated by the ubiquitin/proteasome pathway and involves selective degradation of their C-terminal region containing ankyrin repeats. Whereas the generation of p52 from p100 is a tightly-regulated process, p50 is produced from constitutive processing of p105 [16]. Additionally, the ubiquitin proteasome system controls NF-κB activation also by the degradation of inhibitory proteins, including IκBs members, but there is evidence for different downstream level of NF-κB regulation that employs several non proteolytic mechanisms, including promoter-specific exchange of dimers and modification of the transactivating p65 subunit by post translational modification, such as phosphorylation, acetylation and ubiquitination [17].

In vertebrates, NF-κB is activated by over 150 different stimuli, such as stress, cytokines, free radicals, ultraviolet irradiation, oxidized LDL and bacterial or viral antigens [12]. In turn, there is evidence that active NF-κB participates in the control of transcription of more than 400 genes [13]. These genes include cytokines, chemokines and their modulators, immunoreceptors, proteins involved in antigen presentation, cell adhesion molecules, acute phase proteins, stress response proteins, cell-surface receptors, regulators of apoptosis, growth factors, ligands and their modulators, early response proteins, transcription factors and regulators, and enzymes, controlling several phenomena such as inflammation as well as innate and adaptive immune response [16]. Considering the central role of NF-κB in maintaining cellular homeostasis, it is not surprising that dysregulation of its finely tuned modulation has often been linked to the development of several disorders, most of which with an inflammatory component such as cancer, autoimmune diseases, and chronic inflammatory disorders [18], [19].

Twenty-five years of research efforts [20] have not been enough for a sufficient comprehension of NF-κB dynamics [21], and gaining insights into NF-κB and related processes is still a top priority for the understanding of the onset of associated diseases [20].

### Moving from NF-κB pathway to NF-κB interactome network

It is increasingly recognized that the number of components that impinge upon phenotypic outcomes of signal transduction pathways may be higher than that taken into consideration from canonical representations [22]–[24]. Indeed, recent screens suggest the participation of hundreds of components, instead of the dozens classically involved in canonical signaling pathway representations [22], proposing a switch from linear or branched signal cascades to networks with complex interdependencies and feedbacks. Moreover, the interpretation of new findings [23] suggests that cell functionality may be based on a single “mega-network with limited isolation” [22] between pathways' elements, modules (i.e. a set of proteins capable of independent functionality) and networks. The difficulty in drawing contours and boundaries of signaling pathways becomes more evident when the study of the system is approached with formal engineering failure analysis [25]. Systems biology approaches may provide crucial clues about the architecture and the logic of the regulation of the NF-κB system and related gene expression [20]. Moreover, comprehensive maps of complex signaling pathways are proving to be important tools to facilitate systems-level study [26], [27].

Signaling crosstalk and combinatorial control of NF-κB pathway are very complex [28]–[31]. In-depth studies often report fragmented knowledge, since they forcedly focus only on a limited part of the puzzle. In this scenario limiting analyses to canonical NF-κB pathway elements may reveal inadequate in unveiling crucial mechanisms or players in the regulation of this system. The aim of this work is to integrate into one single, more comprehensive and less “pathway-centric” [22] picture all the proteins that, on the basis of present knowledge, interact directly or indirectly with the fundamental NF-κB-activation proteins to compose the “NF-κB pathway interactome”. We propose that such approach may give further insights on the contribution of components that would have been neglected in a more canonical perspective. Such a comprehensive approach may provide insights about how specific stimuli trigger particular subsets of NF-κB-target genes [21], or about the regulatory hierarchy that rules the selection of target gene expression [32]. To do this, we mined from multiple sources and integrated in an unitary view manually collected data from literature, binary protein-protein interaction (PPI) data, protein annotation data, and NF-κB downstream genes data. We compiled three different sets of proteins that show evidence of involvement in the upstream regulation process of NF-κB, and one set of downstream genes (and related proteins) which expression appears to be regulated by NF-κB. Starting from these sets, we reconstructed three NF-κB protein interaction networks (PINs), or interactomes, by using PPI data from multiple databases, and then checked for the existence of physical interactions between these upstream protein sets and the downstream set. The resulting interactomes also underwent functional enrichment and network analysis to highlight their composition and structure.

## Materials and Methods

Part of data retrieval, interactome reconstruction and analysis have been performed with the use of the Cytoscape network analysis platform [33]– and following a general workflow also described in [36]. For PPI data and interactome reconstruction, we used the Agile Protein Interaction Database (APID) [37], a comprehensive resource for protein interaction data, automatically accessed by Cytoscape through the dedicated plugin APID2NET [38]. APID integrates in a single web-based tool all known experimentally validated protein-protein interactions from BIND, BioGRID, DIP, HPRD, IntAct and MINT databases. Annotation and pathway data have been collected mainly from the Universal Protein Resource (UniProt) and the Kyoto Encyclopedia of Genes and Genomes (KEGG), accessed through the suites FaTiGO [39] and Babelomics [40], [41]. Additional information on disease pathways have been accessed through DisGeNET [42], and the Pharmacogenomics Knowledge Base PharmGKB [43]. All databases and platforms used here are free and open access. The complete protein lists and relative analysis results are provided in tables S1, S2, S3 and dataset S1. Note that we refer to a “set” as to a list of proteins, and to “interactome” as to a network whose elements are (all or a part of) the proteins of the respective set, enriched with the available protein interaction data. Interactome acronyms are indicated with a capital “I” after the acronym of the respective set.

The network analysis have been performed using the Cytoscape platform and the plugins Networkanalyzer [44] and MCODE [45]. Besides classical parameters (node degree and betweenness centrality, among others), we also calculated the network density (the average number of neighbors for each node), and the network centralization. The degree of a node is the number of edges linked to it. In this case, it is the number of physical interactions that a specific protein shows with other proteins. The betweenness centrality of a node attests the grade of control that this node exerts over the interactions of other nodes in the network, and its biological relevance has been demonstrated [46], [47]. The average number of neighbours (connectivity) of a node in the network may also be represented by means of a normalized version of this parameter, i.e. the network density. The density is a value between 0 and 1, and shows how densely the network is populated with links. In calculating density, self-loops are ignored (self-loops are evidence based interactions between two copies of the same protein [44]). Network centrality is a parameter which value ranges between 0 and 1. Networks whose topologies resemble a star (i.e. with a relatively well defined central core) have a centrality close to 1, whereas decentralized networks are characterized by having a centrality close to 0. The interactomes are completely available in Cytoscape format (.cys) in the Dataset S1.

### Directly Interacting (DI) NF-κB proteins dataset

**Figure 1:**
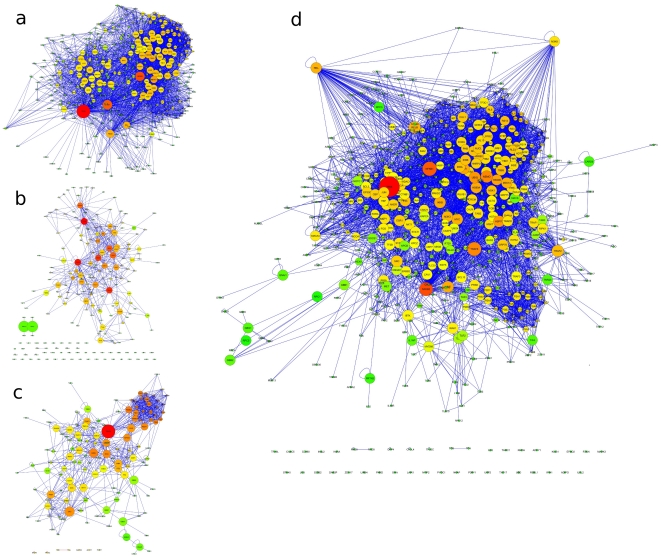
The three interactomes DI (a), UI (b) and MCI (c) and the resulting UNION interactome (d) as from Cytoscape layout, available in the Dataset S1 as .*cys* file. Datasets and interactomes have been reconstructed following procedures described in materials and methods section. Nodes represent proteins and links are evidence-based physical interactions. Node size and color are proportional to betweenness centrality values (red: high, green: low; not comparable among different interactomes). Isolated proteins show no evidence of physical interactions with any other proteins within the same dataset.

The directly-NF-κB-interacting protein set, or hereinafter Direct Interactome, (DI) (fig. 1a), is composed by all the proteins that show experimental evidence of physical interaction with at least one of the five NF-κB members. Given the procedure used (search for directly NF-κB-interacting proteins), in the case of DI, the protein set coincides with the respective interactome. Data retrieval has been performed with APID2NET and Cytoscape. The query returned a total of 377 proteins (including the five NF-κB members). The DI accounts for 4,119 non-directional interactions (including self-interactions).

### Uniprot (U) NF-κB annotated proteins dataset

To populate the set U, we searched the UniProt Knowledge Base with the parameters: *«annotation:(type: nonpositional “nf kappa b”) and organism: “Homo sapiens (Human) [9606]”»* and we retrieved a list of 235 proteins that have been manually screened and checked to obtain a final, validated list of 229 proteins with evidence of implications in the NF-κB functioning (see Table S1 for description). Starting from this set, experimental protein interaction data are retrieved in order to reconstruct the relative interactome. Two hundred and ten proteins (210), out of 229, are present in the APID database, from which interaction data have been downloaded, and constitute the Uniprot interactome (UI, fig. 1b). One hundred and fifty proteins (out of 210) form a main cluster, accounting for 550 interactions, while other 60 are isolated from the main cluster.

### Manually Curated (MC) proteins dataset

We selected and then manually screened a collection of 37 top quality, highly cited literature papers regarding NF-κB to identify proteins that take part with different roles and functions to the signalling cascade leading to NF-κB activation [14], [17], [48]–[82]. The criteria for protein selection and inclusion in the MC set are based on its presence and function described in each paper, either as directly involved in the cascade dynamics (e.g. interacting protein), or collaterally participating with a function identified and described in the articles considered. One hundred and forty one proteins (141) have been identified. Again, PPI data have been added (through the use of the APID database) to build the Manually Curated interactome (MCI), that accounts for 853 non-directional interactions (including self-interactions) (fig. 1c). All proteins in the DI, U and MC sets are considered to work upstream NF-κB, *i.e.* to participate with a variable role to the modulation and to the signalling cascade leading to NF-κBactivation.

### Downstream Genes (DG) dataset

Data extracted from a manually curated list of NF-κB-downstream genes (www.nf-kb.org; [13]) and from the Transcriptional Regulatory Elements Database (TRED; [83], [84]) constitute a list of 441 genes that result to be up- or down-regulated in response to the activation of at least one of the NF-κB family members (Table S2). Four hundred forty one (441) valid ENSEMBL gene identifiers mapping to 426 related protein unique identifiers (Uniprot IDs) have been obtained using online ID converter tools [85]. The Babelomics suite for functional analysis identified 16 duplicates leading to a valid list of 410 IDs. Three hundred and eighty four (384) out of 426 protein IDs have been found in the APID database, used for DG interactome reconstruction.

## Results

### Composition and analysis of the protein sets and interactomes

**Figure 2:**
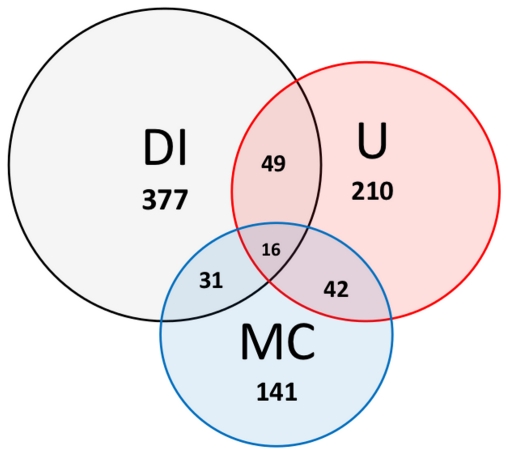
Datasets DI, U and MC share a relatively low number of proteins, as reported by the figures in the intersections. Datasets are quite differentiated in their composition and share only the 2.6% of the whole UNION set (16 out of 622).

The three sets DI, U and MC (and corresponding interactomes DI, UI and MCI) show substantial differences with one another in their dimensions and structure as well as in the protein composition. We also considered the interactome resulting from the union of DI, UI and MCI (DI ∪ UI ∪ MCI; hereinafter UNION, Figure 1d), which accounts for 622 proteins and 6,115 interactions (see table 1 for the main parameters) in order to have the widest possible picture of the elements participating in the NF-κB system. The intersection of all three sets (the proteins shared by all three sets: DI ∩ UI ∩ MCI) accounts for only 16 proteins and 89 interactions (see fig. 2 and Dataset S1). The intersection should represent the very “core” of the system. Indeed it contains the 5 members of the NF-kB family (p65, RelB, c-Rel, NF-κB1, NF-κB2), the 4 members of the inhibitor of NF-κB (IκB) family (IκBα, IκBβ, IκBε, BCL-3) and the three core subunits of the IκB kinase (IKK) complex (NEMO, IKKα, IKKβ) as reported by Perkins [14]. Other components of this core subset are IKBKE, MAP3K14, TRAF3 and NKRF, which are very well known elements able to activate or inhibit NF-κB [86]–[89]. The MCI is the most dense and less populated by self-loops, while the UNION shows high average neighbours value but also many self-loops (Table 1). The most centralised interactome is the DI, while the UI is the most decentralized (Table 1).

**Table 1:** summary of main network parameters of NF-κB upstream protein interactomes DI, UI, MCI and UNION and overrepresented KEGG pathways (p<0.05).

*Interactome*	*No of proteins*	*No of interactions*	*Avg* *neigbours/density*	*Centraliz*	*Main overrepresented KEGG pathways*	*% of pathway covered*	*% of interactome*
**DI**	377	4119	21.1/0.056	0.591	hsa03010 Ribosome	28.8	7.2
					hsa03050 Proteasome	21.8	3.2
					hsa04623 Cytosolic DNA-sensing pathway	21.4	3.2
					hsa04622 RIG-I-like receptor signaling pathway	19.7	4.0
					hsa05215 Prostate cancer	18.9	4.5
					hsa04621 NOD-like receptor signaling pathway	18.9	3.5
					hsa04662 B cell receptor signaling pathway	18.7	3.7
					hsa04920 Adipocytokine signaling pathway	18.0	3.7
					hsa04620 Toll-like receptor signaling pathway	17.6	5.0
					hsa03020 RNA polymerase	17.1	1.6
**U**	210	568	4.6/0.022	0.185	hsa04623 Cytosolic DNA-sensing pathway	40.0	9.6
					hsa04622 RIG-I-like receptor signaling pathway	32.0	10.5
					hsa04621 NOD-like receptor signaling pathway	29.4	8.7
					hsa04620 Toll-like receptor signaling pathway	26.2	12.2
					hsa03020 RNA polymerase (22.9)	22.9	3.5
					hsa04210 Apoptosis	18.5	7.4
					hsa04662 B cell receptor signaling pathway	17.6	5.7
					hsa04920 Adipocytokine signaling pathway	16.9	5.7
					hsa05222 Small cell lung cancer	14.4	5.7
					hsa04660 T cell receptor signaling pathway	13.2	6.6
**MC**	141	853	11.0/0.078	0.232	hsa03050 Proteasome	54.0	19.2
					hsa04210 Apoptosis	27.2	17.7
					hsa04662 B cell receptor signaling pathway	21.6	11.4
					hsa04620 Toll-like receptor signaling pathway	20.6	15.6
					hsa04622 RIG-I-like receptor signaling pathway	20.0	10.6
					hsa04621 NOD-like receptor signaling pathway	19.1	9.2
					hsa05222 Small cell lung cancer	17.8	11.4
					hsa04920 Adipocytokine signaling pathway	16.9	9.2
					hsa04660 T cell receptor signaling pathway	16.7	13.5
					hsa04722 Neurotrophin signaling pathway	16.7	14.9
**UNION**	622	6115	18.9/0.030	0.360	hsa03050 Proteasome	62.0	5.0
					hsa04621 NOD-like receptor signaling pathway	40.6	4.5
					hsa04620 Toll-like receptor signaling pathway	38.0	6.6
					hsa04623 Cytosolic DNA-sensing pathway	37.5	3.4
					hsa03020 RNA polymerase	34.3	1.9
					hsa04622 RIG-I-like receptor signaling pathway	34.2	4.2
					hsa04210 Apoptosis	33.3	5.0
					hsa04662 B cell receptor signaling pathway	30.7	3.7
					hsa05222 Small cell lung cancer	29.7	4.3
					hsa03010 Ribosome	28.7	4.3

See main text for further comments and explanations. Complete datasets available in Table S3.

Network analysis showed that, as expected, being these networks centred on NF-κB, the five NF-κB subunits are among the most central elements for betweenness centrality values (Table 2). This condition is common to all 3 interactomes. The betweenness centrality of a node attests the amount of control exerted by this node over the capability of interaction of other nodes in the network. In particular, four out of five NF-κB components are in the first five positions for betweenness centrality in the UNION set (table 2; complete list available in Dataset S1), while REL is ranked 21^st^. Among the highest ranking proteins, TRAF6, at third position, is the element of connection of crucial membrane receptors. It is a member of the TNF receptor associated factor (TRAF) protein family, and mediates signal transduction from members of the TNF receptor superfamily and of the Toll/IL-1 family [90]. The connection between these two receptor families is interesting because the Toll-Like Receptors (TLRs) and TNF receptors (TNFRs) play the role of external sensors for NF-κB pathway activation. TLRs take a crucial part in early host defense against invading pathogens, recognizing many pathogen-associated molecular patterns (PAMPs), including bacterial cell-surface lipopolysaccharides, peptidoglycans and lipoproteins, viral double-stranded RNA and viral single-stranded RNA, bacterial and viral CpG, as recently reviewed [91]; TNFR is the mediator of TNF effects, one of the universal effectors of innate signaling, involved in host defense and inflammation [92].

**Table 2:** First 25 most central proteins (betweenness centrality) in UNION interactome (in bold the NF-κB components).

Rank	Protein ID	Betwn Centr	Protein description	NCBI gene name
1	**TF65**	0.21071004	**Transcription factor p65**	RELA
2	**NFKB1**	0.08342206	**Nuclear factor NF-kappa-B p105 subunit**	NFKB1
3	TRAF6	0.07545745	TNF receptor-associated factor 6	TRAF6
4	**NFKB2**	0.07071487	**Nuclear factor NF-kappa-B p100 subunit**	NFKB2
5	**RELB**	0.0393886	**Transcription factor RelB**	RELB
6	NEMO	0.03913555	NF-kappa-B essential modulator	IKBKG
7	TRAF2	0.03567854	TNF receptor-associated factor 2	TRAF2
8	SRC	0.03049217	Proto-oncogene tyrosine-protein kinase Src	SRC
9	UBIQ	0.02940406	Ubiquitin	RPS27A
10	IKKB	0.02905882	Inhibitor of nuclear factor kappa-B kinase subunit beta	IKBKB
11	HSP7C	0.02826285	Heat shock cognate 71 kDa protein	HSPA8
12	IKKE	0.02712651	Inhibitor of nuclear factor kappa-B kinase subunit epsilon	IKBKE
13	MYC	0.0253939	Myc proto-oncogene protein	MYC
14	HS90A	0.02399683	Heat shock protein HSP 90-alpha	HSP90AA1
15	ESR1	0.02195341	Estrogen receptor	ESR1
16	IKKA	0.02094338	Inhibitor of nuclear factor kappa-B kinase subunit alpha	CHUK
17	BTK	0.0202242	Tyrosine-protein kinase BTK	BTK
18	IRAK1	0.01966103	Interleukin-1 receptor-associated kinase 1	IRAK1
19	M3K3	0.01862895	Mitogen-activated protein kinase kinase kinase 3	MAP3K3
20	P53	0.0165347	Tumor suppressor gene p53	TP53
21	**REL**	0.01543792	**C-Rel proto-oncogene protein**	REL
22	RIPK2	0.01533207	Receptor-interacting serine/threonine-protein kinase 2	RIPK2
23	BCL10	0.01434518	B-cell lymphoma/leukemia 10	BCL10
24	IKBA	0.01433697	NF-kappa-B inhibitor alpha	NFKBIA
25	STAT3	0.01373161	Signal transducer and activator of transcription 3	STAT3

Table 2 also shows that three broad activators of gene transcription such as Myc, p53 and STAT3 are found as central elements at position 13, 20 and 25, respectively. This reflects the fact that NF-κB is not only important for linking adaptive and innate immunity but it is also at the very centre of fundamental cell defense mechanisms such as stress response, as well as of other basic cellular functions such as survival and proliferation, thus confirming its primordial role as central actor in the protection of a multicellular organism's integrity [21]. Interestingly, the highly conserved protein UBIQ (ubiquitin) ranks at position 9 (Table 2), while other well-known inhibitors of NF-κB, IKKB, IKKE, IKKA and IKBA rank at position 10, 12, 16 and 24, respectively. The presence at such top ranking positions of factors that inhibit NF-κB or target it to proteasomal degradation is a further indication of the centrality of this complex, which, due to its pleiotropic effects, must be tightly controlled [93].

We observed a limited degree of overlapping among the sets (Fig. 2): DI and U share only the 9.1% of their elements, DI and MC the 6.4%, U and MC the 13.6%, while only the 2.6% of the elements are shared by all three interactomes (16 out of 622). These numbers partly reflect the difference and dishomogeneity in sources, databases, data types and relative retrieval method that have been used. Nevertheless, we did not expect such remarkable discrepancy in the composition of the sets, discrepancy that might not be totally explained simply taking into account the wide differences in data sources and the integrative approach used, but, on the contrary, should cast some doubts about the adequacy and the completeness of the classical pathway descriptions.

Another example of such inadequacy is the fact that from the analysis of the datasets TAK1 (MAP3K7) did not result to be high ranking, as it would be expected [94].

By using the Babelomics platform, we analyzed all the sets to identify the metabolic or signalling pathways which elements are shared with the given sets. In Table 1 we show the first ten over-represented pathways in the DI, U, MC and UNION according to this analysis. The complete lists of KEGG significant terms (p-value<0.05) are available in the Table S3. We noted that 5 KEGG pathways resulted as ranking within the top 10 in DI, U, and MC, and that 4 out of 5 are pathways linked to the induction of innate immunity response and inflammation (Toll-like receptor signaling pathway; RIG-I-like receptor signalling pathway; NOD-like receptor signalling pathway; Adipocytokine signalling pathway). This confirms that, even if the sources of these sets are very different, our analysis was able to capture the role of NF-κB as mediator of inflammation. Interestingly, MAPK pathway ranks always high in terms of percentage of proteins present in all three protein sets, indicating that also signal transduction leading to NF-κB activation is a process whose component are captured by our analysis. The UNION set resumes the characteristics of the three and represents, in number of components, 62% of the whole KEGG-annotated proteasome and 41% of the NOD signalling pathway, among others.

### Downstream gene and protein set: composition and characteristics

The same KEGG analysis has been done on NF-κB downstream gene set (410 out of 426 identifiers have been recognized by the Babelomics analysis suite). Among the top 10 identified pathways (Table 3), four are involved in inflammation (Toll-like receptor signalling pathway; NOD-like receptor signalling pathway; cytosolic DNA-sensing pathway; cytokine-cytokine receptor interaction), with two of them also shared by the DI, U and MC sets. The analysis has also pointed out that relevant subsets of DG are related to pathologies with well-known inflammatory aetiology, such as bladder and prostate cancers, among others.

**Table 3:** Overrepresented pathways in DG (p<0.05), first 20 terms ranked for percentage of DG proteins present in KEGG pathway (“% of set” column).

KEGG ID	Pathway	Total terms in KEGG pathway	Terms in set and pathway	% of set	% of pathway
**hsa04060**	Cytokine-cytokine receptor interaction	271	70	17,07	25,83
**hsa04062**	Chemokine signaling pathway	186	30	7,32	16,13
**hsa04630**	Jak-STAT signaling pathway	154	26	6,34	16,88
**hsa04620**	Toll-like receptor signaling pathway	101	23	5,61	22,77
**hsa05222**	Small cell lung cancer	90	19	4,63	21,11
**hsa04621**	NOD-like receptor signaling pathway	62	17	4,15	27,42
**hsa05215**	Prostate cancer	88	17	4,15	19,32
**hsa04210**	Apoptosis	86	16	3,90	18,60
**hsa04940**	Type I diabetes mellitus	90	16	3,90	17,78
**hsa04672**	Intestinal immune network for IgA production	84	15	3,66	17,86
**hsa05330**	Allograft rejection	84	15	3,66	17,86
**hsa04640**	Hematopoietic cell lineage	87	15	3,66	17,24
**hsa05332**	Graft-versus-host disease	87	13	3,17	14,94
**hsa05219**	Bladder cancer	42	12	2,93	28,57
**hsa04115**	p53 signaling pathway	67	11	2,68	16,42
**hsa05218**	Melanoma	70	11	2,68	15,71
**hsa05212**	Pancreatic cancer	71	11	2,68	15,49
**hsa04623**	Cytosolic DNA-sensing pathway	55	10	2,44	18,18
**hsa05340**	Primary immunodeficiency	41	9	2,20	21,95
**hsa05014**	Amyotrophic lateral sclerosis	57	9	2,20	15,79

**Figure 3:**
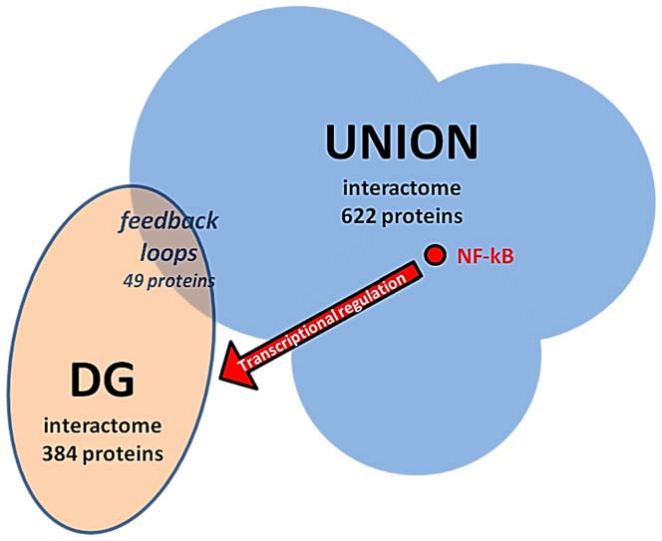
The UNION interactome is composed by 622 proteins, including the five NF-κB subunits. NF-κB is able to regulate the expression of 426 proteins (DG set, see main text). A subset (384 proteins, present in the APID database, out of 426) has been checked for PPIs (see Dataset S1). Forty nine proteins are shared by both the UNION and the DG sets, establishing a feedback loop: “*interaction with NF-κB pathway → transcription factor activation → transcriptional regulation → interaction with NF-κB pathway*”, meaning that 13% of the identified NF-κB-regulated genes express proteins that play a direct role in the UNION interactome.

We noted that 49 proteins are present in both UNION and DG sets (Table 4), thus meaning that 13% of the identified NF-κB-regulated genes express proteins that play a direct role in the UNION interactome (Figure 3). These data suggest that the implication of such feedback loop in the dynamics of the interactome and in its regulation and activation of NF-κB may be remarkable. The ability of NF-κB to control the transcription of a non negligible part of its activation pathway interactome surely deserves a deeper attention. Since NF-κB is a constitutively expressed protein complex, and since it has a crucial importance in the regulation of fundamental tasks such as the immune responses and inflammation, it must be tightly self-regulated to prevent malfunctions and inappropriate activation.

**Table 4:** “Feedback” genes and related proteins (protein ID alphabetical order).

Protein ID	Protein Description	NCBI gene name
ANDR	Androgen receptor	AR
BCL3	B-cell lymphoma 3-encoded protein	BCL3
BIRC4	Baculoviral IAP repeat-containing protein 4	BIRC4
BLNK	B-cell linker protein	BLNK
BTK	Tyrosine-protein kinase BTK	BTK
CAV1	Caveolin-1	CAV1
CFLAR	CASP8 and FADD-like apoptosis regulator	CFLAR
EGR1	Early growth response protein 1	EGR1
ELF3	ETS-related transcription factor Elf-3	ELF3
ERBB2	Receptor tyrosine-protein kinase erbB-2	ERBB2
GCR	Glucocorticoid receptor	NR3C1
HS90A	Heat shock protein HSP 90-alpha	HSP90AA1
IKBA	NF-kappa-B inhibitor alpha	NFKBIA
IKBE	NF-kappa-B inhibitor epsilon	NFKBIE
IKBZ	NF-kappa-B inhibitor zeta	NFKBIZ
IL2RA	Interleukin-2 receptor alpha chain	IL2RA
IL32	Interleukin-32	IL32
IRF1	Interferon regulatory factor 1	IRF1
IRF2	Interferon regulatory factor 2	IRF2
KISS1	Metastasis-suppressor KiSS-1	KISS1
LYSC	Lysozyme C	LYZ
MYC	Myc proto-oncogene protein	MYC
**NFKB1**	Nuclear factor NF-kappa-B p105 subunit	NFKB1
**NFKB2**	Nuclear factor NF-kappa-B p100 subunit	NFKB2
NOD2	Nucleotide-binding oligomerization domain-containing protein 2	NOD2
NUAK2	NUAK family SNF1-like kinase 2	NUAK2
OLR1	Oxidized low-density lipoprotein receptor 1	OLR1
P53	Tumor suppressor gene p53	TP53
PRGR	Progesterone receptor	PGR
PSA2	Proteasome subunit alpha type-2	PSMA2
PSB9	Proteasome subunit beta type-9	PSMB9
PTEN	Phosphatidylinositol-3,4,5-trisphosphate 3-phosphatase PTEN	PTEN
**REL**	C-Rel proto-oncogene protein	REL
**RELB**	Transcription factor RelB	RELB
RIPK2	Receptor-interacting serine/threonine-protein kinase 2	RIPK2
TCAM1	TIR domain-containing adapter molecule 1	TICAM1
TERT	Telomerase reverse transcriptase	TERT
TGM2	Protein-glutamine gamma-glutamyltransferase 2	TGM2
TIFA	TRAF-interacting protein with FHA domain-containing protein A	TIFA
TLR2	Toll-like receptor 2	TLR2
TLR9	Toll-like receptor 9	TLR9
TNAP3	Tumor necrosis factor, alpha-induced protein 3	TNFAIP3
TNF15	Tumor necrosis factor ligand superfamily member 15	TNFSF15
TNIP1	TNFAIP3-interacting protein 1	TNIP1
TNIP3	TNFAIP3-interacting protein 3	TNIP3
TRAF1	TNF receptor-associated factor 1	TRAF1
TRAF2	TNF receptor-associated factor 2	TRAF2
TWST1	Twist-related protein 1	TWIST1
VIME	Vimentin	VIM

NF-κB controls the transcription of hundreds of genes. Forty-nine of them code for proteins that belong to the UNION interactome, i.e. that participate in the activation of NF-κB. The circuit “interaction with NF-κB pathway → transcription factor activation → transcriptional regulation → interaction with NF-κB pathway” establishes a number of feedback loops that can play a role in the dynamics of the NF-κB system. In **bold** the four NF-κB components which transcription is regulated by NF-κB itself.

Several feedback loops are already well-known and studied in the literature, while many others have not been taken under consideration in the perspective of the possible implications in the regulation of the NF-κB pathway dynamics. In fact, as already reported in literature and confirmed by our analysis (Table 4), NF-κB complexes directly control the expression of their own subunits with the exception of TF65 [14]. Thus, NF-κB system appears to be very tightly self-regulated.

## Discussion

As mentioned in the introduction, an increase of inflammatory markers has been described as a general feature of the ageing process and proposed as a possible cause of many age-associated diseases. Therefore, the system of NF-κB is likely a crucial player in this process. In this perspective, in order to understand how the changes in composition and abundance of the NF-κB interactome can regulate NF-κB activation, and thus (at least in part) the ageing process, the knowledge about NF-κB pathway as well as the proteins that can interact directly or indirectly with it must be broaden. To this purpose, we integrated data from multiple existing sources in order to chart the NF-κB interactome map. Our map may help in improving the understanding of complex signalling networks and highlights the impact of single elements, their feedback and crosstalk regulations on cellular processes [26]. Beside confirming previous studies and insights [22], the integrative effort proposed here in merging existing, freely available data highlights that the number of elements impinging upon NF-κB-activating pathway outcomes is much higher than that usually taken under consideration in canonical pathway representations.

Furthermore, as other pleiotropic factors that receive many inputs and spread out many outputs, NF-κB core elements are at the centre of a network characterised by a bow tie architecture [95], with a fan in (UNION interactome), a knob (the NF-κB family), a fan out (downstream genes modulated by NF-κB) and feedback loops. This is not surprising, since the bow tie might represent an economical and efficient way to convey on the same target different stimuli to which cells and organs are exposed [95], [96]. Moreover, bow tie architecture may be an advantageous solution for the evolution of complex systems, because it would minimize the number of central molecular mediators, ultimately reducing energy expenditure for both the integration of stimuli and the determination of the outcome [97], [98]. Bow ties are considered to be fundamental in explaining the coexistence of robustness and evolvability in complex systems due to their ability to facilitate control [95], [99]–[102]. Another striking feature of bow ties' functioning is their inner resilience characterised by the presence of regulatory feedback loop, *i.e.* some products of the fan out are also part of the fan in. In this case the NF-κB system fits perfectly with this architecture, as we checked for proteins present in both the UNION interactome and DG set, thus establishing a feedback loop of the type: *interaction with NF-κB pathway → transcription factor activation → transcriptional regulation → interaction with NF-κB pathway* (Table 4). Concerning such feedbacks, we report three cases that were correctly captured by our analysis. The Tumor suppressor protein p53 –that appears among the “feedback” proteins- can be transcriptionally induced by NF-κB [103]–[105], among others. On its side, p53 can interfere with NF-κB activity [106], [107] thus creating a sort of negative feedback loop between the two factors. The two factors not only interact with each other, but also cooperate or compete (depending upon circumstances) to activate downstream genes [108], [109]. The analysis also identified a complex cross-regulatory loop involving NF-κB and PTEN, a “feedback” protein, that may serve to balance their mutually antagonistic functions. Indeed, PTEN is down-regulated by NF-κB and is able to negatively regulate NF-κB, establishing a complex regulation which determines cell survival or apoptosis [110], [111]. The “feedback” ubiquitin-editing protein TNAP3 (TNFAIP3) has been described as a key player in the termination of NF-κB signaling and in controlling NF-κB-dependent inflammation [112]. In most cell types basal TNAP3 expression is very low but its transcription is rapidly induced upon NF-kB activation. Once expressed, TNAP3 functions as a negative feedback regulator of NF-κB activation [112].

Based upon these evidences, we propose that a comprehensive and systemic consideration of “feedback” genes may complement important findings in autoregulatory feedback loops [113] leading to a deeper understanding of the complex NF-κB regulation and dependent inflammation. In this perspective, the observation of a number of feedback loops deserves a deeper systemic and experimental investigation, since these interactions might be potential critical points for the NF-κB system regulation neglected so far. In this direction, results from this interactome reconstruction can be used as preliminary screening to identify putative key regulators of NF-κB system.

In summary, substantial divergences in the composition of the DI, U and MC sets open questions about the adequacy and comprehensiveness of classical pathway descriptions and representations, and suggest the participation of a number of proteins one order of magnitude higher than that classically taken into consideration, thus transforming the concept of pathway from an isolated entity into an open, unbound one. The map as it is introduced here provides a tool to explore the complexity of the NF-κB system and to make useful qualitative predictions regarding key regulators and mediators of its activating signalling cascade, but it is only a first step in this direction. The next steps will be not only the obvious and continuous updates of existing records and newly produced data, but also the charting of the interactome of the different NF-κB components, and the analysis of interacting proteins taking into account their sub-cellular compartmentalization, which is currently one of most promising perspective for the elucidation of the mechanisms related to ageing [114]. To this regards, it is also plausible that NF-κB interactome can be differently charted considering as a variable the five NF-κB members, as well as the sub-cellular localisation of its components (e.g. nucleus, organelles, membranes, cytoplasm).

A further important step will consist in charting cell type-specific, and time-dependent interactomes, able to show maps of various cellular functions, given that NF-κB has not only a major role in inflammatory status but it has also a crucial role in neuronal cells, for instance in the Schwann cell myelination, at least in mouse models [115]. Dynamic analysis of interactomes is the next fascinating aim, crucial for the better understanding of protein-protein interactions when data from time-resolved proteomics will be completely available, together with an adequate mathematical and algorithmic contribution [116], [117]. This approach could be extremely relevant to definitively disentangle when, where and how protein-protein interactions give their contribution to the different functional levels of cell systems (different types of cells, tissues, different responses to various stimuli etc). To this regard, network inference based on the huge amount of data gathered in the Omics era might represent a successful approach [118].

As a whole, the results of the integrative approach shown and reported here are meant to be used as a starting point to identify new roles of NF-κB in physiology and diseases. The integrated analysis we performed identified elements and pathways that are not immediately linked to NF-κB itself, or not taken into consideration in respect to its regulation and dynamics, and may thus suggest new directions for further studies and analyses.

## Supporting Information

Table S1 Uniprot annotations retrieved and used to populate protein dataset U.
(XLS)

Table S2 gene identifiers extracted from a manually curated list of NF-κB-downstream genes [13] and from the Transcriptional Regulatory Elements Database (TRED; [83], [84]), and related protein unique identifiers (Uniprot IDs) obtained using online ID converter tools [85] used to compile DG dataset.
(XLS)

Table S3 overrepresented KEGG pathways in DI, U, MC and DG datasets.
(XLSX)

Dataset S1 complete Cytoscape network session with full datasets and interactomes.
(RAR)
